# Prevalence of HER3 Expression in Pancreatic Cancer Patients Treated With Systemic Chemotherapy

**DOI:** 10.1002/cam4.70474

**Published:** 2024-12-09

**Authors:** Tomoyuki Satake, Chigusa Morizane, Mao Okada, Mariko Nishioka, Nobuyoshi Hiraoka, Satoshi Nara, Tomoya Kakegawa, Maki Kobayashi, Kumiko Koyama, Minoru Esaki, Takuji Okusaka

**Affiliations:** ^1^ Department of Hepatobiliary and Pancreatic Oncology National Cancer Center Hospital East Kashiwa Japan; ^2^ Department of Hepatobiliary and Pancreatic Oncology National Cancer Center Hospital Tokyo Japan; ^3^ Division of Pathology and Clinical Laboratories National Cancer Center Hospital Tokyo Japan; ^4^ Department of Hepatobiliary and Pancreatic Surgery National Cancer Center Hospital Tokyo Japan; ^5^ Translational Research Laboratories, Daiichi Sankyo Co. Ltd. Tokyo Japan

**Keywords:** cancer genome profiling, chemotherapy, HER3, immunohistochemistry, pancreatic cancer

## Abstract

**Background:**

Although activation of human epidermal growth factor receptor 3 (HER3) is linked to resistance to targeted therapies in several cancer types, the HER3 expression profile during pancreatic cancer treatment remains unknown.

**Aims:**

We evaluated the HER3 expression status after chemotherapy for pancreatic cancer and its association with clinicopathological features and clinical outcomes.

**Materials & Methods:**

We included patients with pancreatic cancer who underwent chemotherapy and whose post‐treatment archival tissue specimens were collected. HER3 expression was retrospectively assessed by immunohistochemistry scoring (0, 1+, 2+, and 3+) of the membranous staining intensity.

**Results:**

HER3 expression after chemotherapy was evaluated in 41 patients, with matched‐pair analysis in five patients before and after chemotherapy. HER3 expression was observed in most of the patients after chemotherapy, demonstrating IHC scores of ≥ 1+ and ≥ 2+ in 40 (98%) and 26 (63%) of 41 patients, respectively. Of the 38 patients with adenocarcinoma, the median overall survival in the HER3 (2+/3+) and HER3 (0/1+) groups was 21.0 and 17.1 months, respectively. The comparison of HER3 expression before and after chemotherapy performed in five cases revealed that scores changed from 2+/3+ to 0/1+ in one case, 0/1+ to 2+/3+ in another case, and remained at 2+/3+ in three cases. Cancer genome profiling tests in eight cases found no HER3 amplification or mutation, and seven of these cases had adenocarcinomas with *KRAS* and *TP53* mutations.

**Conclusion:**

A high prevalence of HER3 expression was observed in pancreatic cancer patients after chemotherapy. Our findings indicate that HER3 is a potential therapeutic target for pancreatic cancer, deserving further clinical investigation.

## Graphical abstract

We evaluated the HER3 expression status after chemotherapy for pancreatic cancer and its association with clinicopathological features and clinical outcomes. A high prevalence of HER3 expression was observed in pancreatic cancer patients after chemotherapy. Our findings indicate that HER3 is a potential therapeutic target for pancreatic cancer, deserving further clinical investigation.

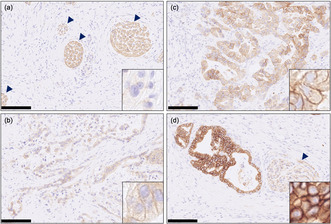

**Abbreviations**
CGP: cancer genome profiling
EGFR: epidermal growth factor receptor
GnP: gemcitabine plus nab‐paclitaxel
HER2: human epidermal growth factor receptor 2
HER3: human epidermal growth factor receptor 3
IHC: immunohistochemistry
NSCLC: non–small cell lung cancer
ORR: objective response rate
OS: overall survival
TKI: tyrosine kinase inhibitor

## 1 Introduction

Human epidermal growth factor receptor 3 (HER3/ErbB3) is a receptor tyrosine kinase that belongs to the HER family and shows little or no intrinsic tyrosine kinase activity [1, 2]. In comparison with other epidermal growth factor receptor (EGFR) family members, HER3 shows differences at critical residues in its intracellular kinase domain, which is locked in an inactive‐like conformation, leading to 1000‐fold weaker kinase activity than that of EGFR [2, 3]. However, HER3 can form heterodimers with other EGFR family members, which is responsible for activating oncogenic signaling pathways [4, 5, 6]. Furthermore, HER3 activates the PI3K/Akt/mTOR signaling pathway for cancer cell survival by directly binding to PI3K [7]. HER3 also activates the MAPK and the JAK–STAT and proto‐oncogene c‐Src signaling pathways for cancer cell proliferation [5, 6, 8, 9].

HER3 expression is associated with disease progression and metastasis in various types of cancer [10, 11]. Two systematic analyses across multiple malignant tumor types, including pancreatic cancer, confirmed that HER3 expression was associated with worse overall survival and a 1.6‐fold higher risk of death than that in HER3‐negative patients [12, 13]. HER3 expression also serves as a bypass mechanism for various therapies, and elevated HER3 signaling confers resistance to multiple therapeutic agents [9, 14, 15, 16, 17, 18, 19, 20]. HER3 expression is reported to be potentially altered before and after systemic therapy. HER3 expression levels are high in NSCLC with EGFR mutations, and EGFR inhibition increases HER3 expression [21]. HER3 is considered to be a target for anticancer treatment [22]. The variation in HER3 expression before and after systemic therapy is also important from the perspective of drug development.

Pancreatic cancer is one of the most aggressive tumors and the fourth‐leading cause of cancer‐related deaths in the United States and Japan [23, 24]. Patients with this disease have an extremely poor prognosis, and the US “Cancer Statistics, 2023” reported that the 5‐year relative survival rate for pancreatic cancer was 12% for all stages and only 3% for metastatic disease (the most common form) [23]. Pancreatic cancer is expected to become the second‐leading cause of cancer‐related deaths by 2030 [25]. Therefore, novel therapies with improved efficacy are urgently needed to improve the prognosis of patients with this disease.

Only a few reports on HER3 expression in pancreatic cancer have been published to date, and little is known about the clinical importance of HER3 expression in pancreatic cancer patients. Given the hypothesis that HER3 expression after chemotherapy may be different from that previously reported at the time of diagnosis, we investigated the status of HER3 expression after chemotherapy for pancreatic cancer and evaluated the associations among HER3 expression, clinicopathological features, and patient clinical outcomes.

## 2 Materials and Methods

### 2.1 Patients

We retrospectively reviewed the medical records of patients with pancreatic cancer treated at the National Cancer Center Hospital between January 2010 and June 2020. Only patients who had previously undergone chemotherapy and for whom post‐chemotherapy tissue samples collected after or during the treatments were available were included in this study. Patients currently undergoing treatment or follow‐up at our institution were excluded from this study to avoid any clinical disadvantages of obtaining tissue specimens. Clinical and histopathological characteristics of the patients, including age, sex, ECOG performance status, histopathology type, primary site of the pancreatic tumor lesion (head, body, tail), disease stage at diagnosis (resectable, locally advanced, metastatic), tumor size, tissue collection method, chemotherapy regimen, and carcinoembryonic antigen and carbohydrate antigen 19‐9 levels at tissue collection, were collected and analyzed. Tumor response was analyzed using the Response Evaluation Criteria in Solid Tumors version 1.1 [26]. Data were retrospectively collected from medical chart reviews and electronic records. This study was approved by the Institutional Review Board of the National Cancer Center, Tokyo, Japan (Approval Number: 2018‐149). Written informed consent had been obtained from all participants.

### 2.2 Immunohistochemical Staining and Evaluation

HER3 membrane expression was assessed by immunohistochemistry (IHC) in pre‐ and post‐chemotherapy tissue samples using previously reported methods [27]. Briefly, HER3 IHC was performed on 4‐μm‐thick sections prepared from paraffin blocks of 10% neutral buffered formalin‐fixed specimens. Sections of each sample were deparaffinized, and antigen retrieval was performed using a PT Link machine (Dako; Agilent Technologies Inc.) at high pH. Sections were stained with a rabbit monoclonal antibody against HER3/ErbB3 (1:59 dilution; clone D22C5; Cell Signaling Technology Inc.) using the Dako autostainer Link48 (Dako) and EnVision Flex Mini Kit (Dako) according to the manufacturer's instructions. The slides were counterstained with hematoxylin. HER3 expression was evaluated as IHC scores of 0 (no staining), 1+ (weak staining), 2+ (moderate staining), and 3+ (strong staining).

### 2.3 Analysis of Genomic Alterations

We analyzed genomic alterations in eight patients who had undergone testing for genomic alterations under normal medical care and analyzed the findings in relation to HER3 expression. In Japan, three types of next‐generation sequencing‐based cancer genome profiling (CGP) tests—OncoGuide NCC Oncopanel System, FoundationOne CDx, and FoundationOne Liquid CDx—are reimbursed by the national health insurance system and implemented in routine oncological practice. Only genetic mutations reported as “pathogenic” or “likely pathogenic” were included in the analysis.

### 2.4 Statistical Analysis

Overall survival (OS) was defined as the period from the start of chemotherapy (neoadjuvant therapy or first‐line chemotherapy) to death or last follow‐up. OS was estimated using the Kaplan–Meier method and compared between independent groups using the log‐rank test. Statistical significance was set at *p* < 0.05. Statistical analysis was performed using EZR software version 1.38 (Saitama Medical Center, Jichi Medical University, Saitama, Japan) [28].

## 3 Results

### 3.1 Patient Characteristics

**FIGURE 1:**
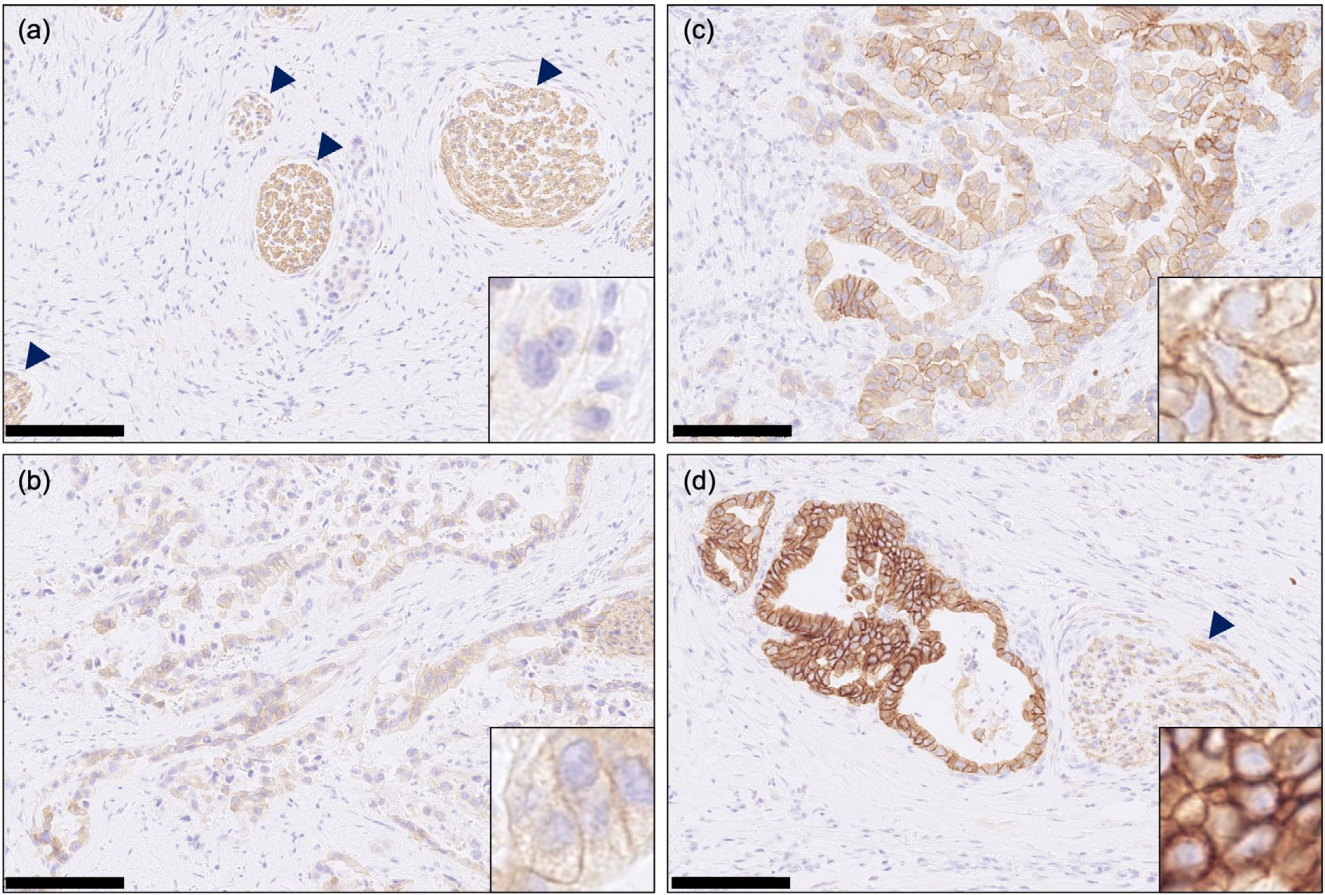
Representative images of HER3 immunohistochemical (IHC) staining quantified as (a) HER3 IHC score 0, (b) HER3 IHC score 1, (c) HER3 IHC score 2, and (d) HER3 IHC score 3 in post‐chemotherapy specimens. The scale bar shown at the bottom left of the image indicates 100 μm. Insets are ×80 magnification images. The arrowheads indicate the nerve fibers as an internal positive control for HER3 IHC.

**FIGURE 2:**
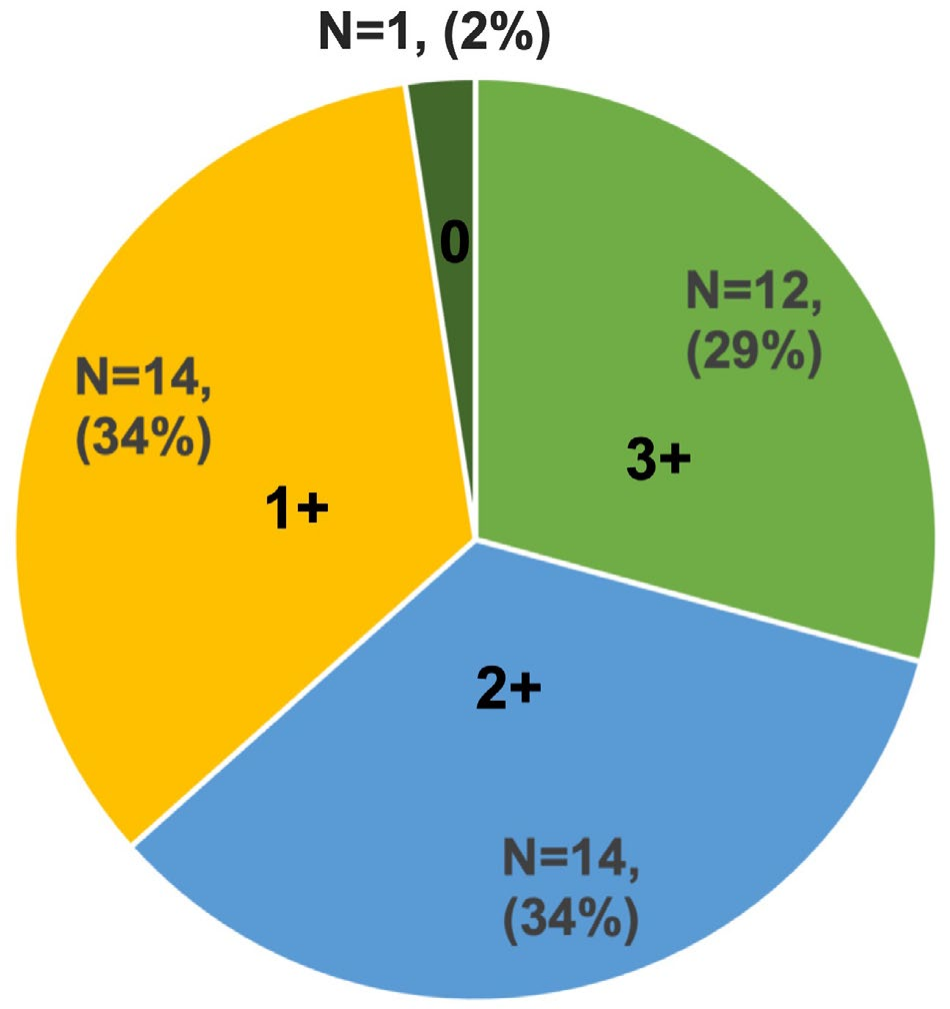
Distribution of HER3 expression by immunohistochemistry.

A Total of 41 patients who were eligible for HER3 expression analysis after chemotherapy were included in this study (Figure S1 and Table S1). In addition, for five patients, pre‐chemotherapy tissue samples were available in sufficient amounts for HER3 expression analysis. Of 41 post‐chemotherapy tissue samples, HER3 expression IHC scores ≥ 1+ were observed in 40 patients (98%), while HER3 scores ≥ 2+ were observed in 26 patients (63%) (Figures 1 and 2). Clinicopathological features of 41 patients are summarized in Table 1, where patients were divided, on the basis of their HER3 expression status, into two groups: 2+/3+ and 0/1+. Overall, the primary site was the pancreatic head in 68% of the patients, and 93% of the patients were diagnosed as showing adenocarcinoma on histopathological assessments. Specimens were mainly obtained by surgery (81%), while a few were obtained by biopsy (19%). There was no major difference in patient background between the HER3 (2+/3+) group and the HER3 (0/1+) group, but the median tumor size of the primary lesion was 30 mm in the HER3 (2+/3+) group and 35 mm in the HER3 (0/1+) group. Approximately 40% of the patients showed a locally advanced stage at diagnosis in both groups; 46% of the tumors were resectable and 12% were metastatic in the HER3 (2+/3+) group, and 20% were resectable and 33% were metastatic in the HER3 (0/1+) group. Chemotherapy administered prior to specimen collection was often gemcitabine plus S‐1 for patients with resectable disease and gemcitabine plus nab‐paclitaxel (GnP) or FOLFIRINOX (oxaliplatin, irinotecan, leucovorin, and fluorouracil) for patients with locally advanced disease or distant metastases. With respect to differences in HER3 expression by chemotherapy regimen, GnP showed HER3 (2+/3+) in all 5 patients, followed by GEM+S‐1 and FOLFIRINOX, which showed HER3 (2+/3+) in 73.3% and 50% of patients, respectively (Table S2).

**TABLE 1:** Clinicopathological variables in HER3 (2+/3+) group and HER3 (0/1+) group.

	Overall	HER3 high (IHC 2+/3+)	HER3 low (IHC 0/1+)
	*N* = 41	*N* = 26	*N* = 15
Age, years			
Median (range)	66 (48–82)	68 (48–82)	63 (57–79)
Sex, *n* (%)			
Male	22 (53.7%)	14 (53.8%)	8 (53.3%)
Female	19 (46.3%)	12 (46.2%)	7 (46.7%)
ECOG PS, *n* (%)			
0	29 (70.7%)	18 (69.2%)	11 (73.3%)
1	12 (29.3%)	8 (30.8%)	4 (26.7%)
Histopathologic classification, *n* (%)			
Adenocarcinoma	38 (92.7%)	24 (92.3%)	14 (93.3%)
Well differentiated	2	2	0
Moderately differentiated	21	13	8
Poorly differentiated	8	6	2
Not evaluable	7	3	4
Others^a^	3 (7.3%)	2 (7.7%)	1 (6.7%)
Primary lesion, *n* (%)			
Head	28 (68.3%)	18 (69.2%)	10 (66.7%)
Body	8 (19.5%)	6 (23.1%)	2 (13.3%)
Tail	5 (12.2%)	2 (7.7%)	3 (20.0%)
Disease stage at diagnosis, *n* (%)			
Resectable	15 (36.6%)	12 (46.2%)	3 (20.0%)
Locally advanced	18 (43.9%)	11 (42.3%)	7 (46.7%)
Metastatic	8 (19.5%)	3 (11.5%)	5 (33.3%)
Tumor size (mm)			
Median (range)	32.5 (10–79)	30 (15–72)	35 (10–79)
Tissue collection, *n* (%)			
Surgery	33 (80.5%)	22 (84.6%)	11 (73.3%)
Biopsy	8 (19.5%)	4 (15.4%)	4 (26.7%)
Treatment, *n* (%)			
FOLFIRINOX	12 (29.3%)	6 (23.1%)	6 (40.0%)
GEM+nab‐PTX	5 (12.2%)	5 (19.2%)	0 (0.0%)
GEM+S‐1	15 (36.6%)	11 (42.3%)	4 (26.7%)
Chemoradiotherapy	4 (9.8%)	1 (3.8%)	3 (20.0%)
Others^b^	5 (12.2%)	3 (11.5%)	2 (13.3%)
CEA (ng/mL) at tissue collection, *n* (%)			
≦ ULN	32 (78.0%)	20 (76.9%)	12 (80.0%)
> ULN	9 (22.0%)	6 (23.1%)	3 (20.0%)
CA19‐9 (U/mL) at tissue collection, *n* (%)			
≦ ULN	12 (29.3%)	8 (30.8%)	4 (26.7%)
> ULN	29 (70.7%)	18 (69.2%)	11 (73.3%)

^a^ Adenosquamous cell carcinoma (*n* = 1), anaplastic carcinoma (*n* = 1), acinar cell carcinoma (*n* = 1).

^b^ S‐1 (*n* = 2), pembrolizumab (*n* = 1), trametinib (*n* = 1), GEM (*n* = 1).

### 3.2 Association of HER3 Expression After Chemotherapy With Overall Survival

**FIGURE 3:**
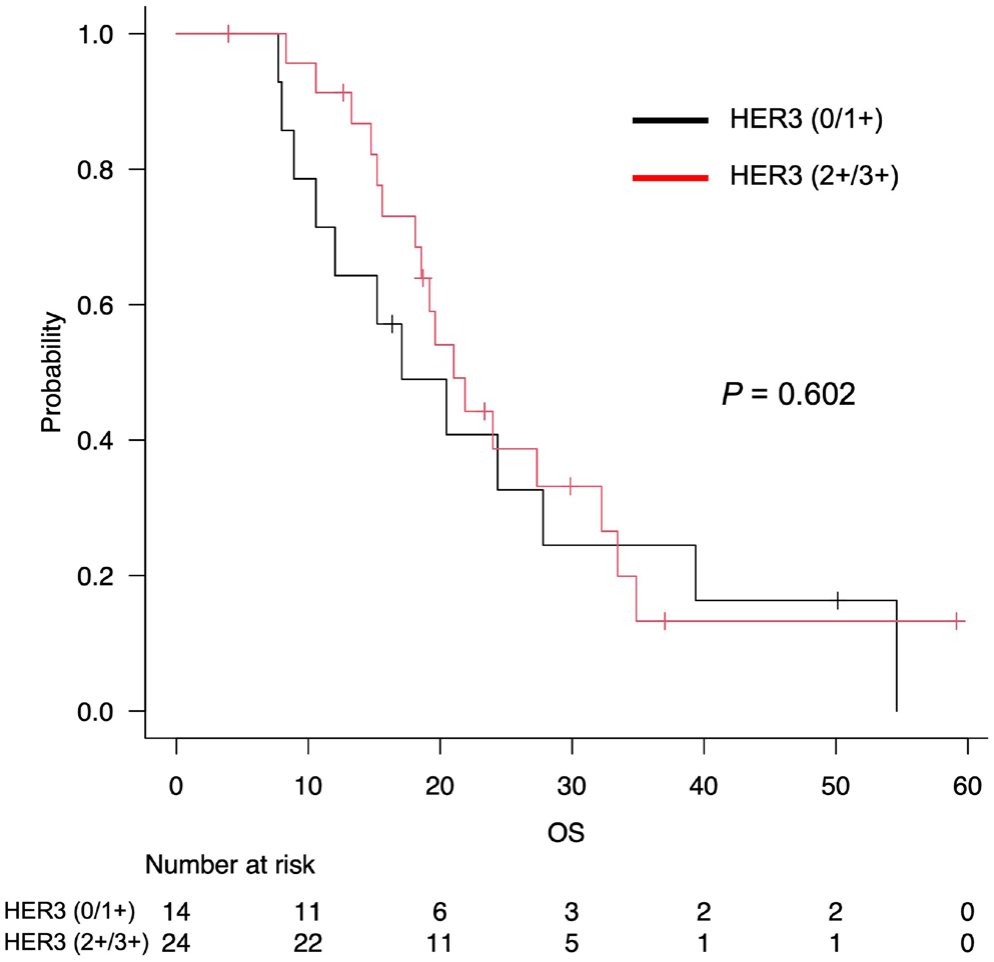
Kaplan–Meier survival analysis of overall survival according to HER3 expression.

In 38 cases of adenocarcinoma, excluding three cases of rare subtypes, the median OS was 21.0 months (95% CI: 15.6–32.2) in the HER3 (2+/3+) group and 17.1 months (95% CI: 8.9–27.8) in the HER3 (0/1 +) group. No significant difference in OS (*p* = 0.602) was observed between the HER3 (2+/3+) and HER3 (0/1+) groups (Figure 3). Subgroup analysis by disease stage also showed no significant difference in OS by HER3 expression (Figure S2).

### 3.3 Differences in HER3 Expression Pre‐ and Post‐Chemotherapy

Pre‐chemotherapy HER3 status was analyzed in five cases, allowing the comparison of HER3 expression pre‐ and post‐chemotherapy (Figure S1). Only one case showed an increase in the IHC score for HER3 expression after chemotherapy, while the remaining cases showed no change in the IHC score (one case) or a reduction in the score (three cases; Table 2). When HER3 expression scores were categorized as 2+/3+ or 0/1+, the scores changed from 2+/3+ to 0/1+ in one case, from 0/1+ to 2+/3+ in another case, and remained at 2+/3+ in three cases. Assessments of the efficacy of chemotherapy among these five patients revealed that a partial response according to RECIST 1.1 was observed in three patients, and the carbohydrate antigen 19‐9 (CA19‐9) level was lower than the pretreatment level in four patients. Among the three cases showing partial response, two showed altered HER3 IHC expression (one showed an increase in the score and the other showed a decrease).

**TABLE 2:** Changes in HER3 expression before and after chemotherapy.

	Before chemotherapy		Chemotherapy			After chemotherapy	
Patient	HER3 expression	CA19‐9 (U/mL)	Treatment	Aim of chemotherapy	Best response	HER3 expression	CA19‐9 (U/mL)
P006	3+	1189	FOLFIRINOX	First line for advanced stage	PR	0	61
P020	3+	111	GEM+S‐1	Neo adjuvant	PR	2+	49
P029	1+	1373	FOLFIRINOX	First line for advanced stage	PR	2+	54
P034	3+	39	GEM+S‐1	Neo adjuvant	SD	2+	98
P041	3+	81,610	S‐1	Third line for advanced stage	SD	3+	41,400

### 3.4 HER3 Expression and Genomic Alterations

**FIGURE 4:**
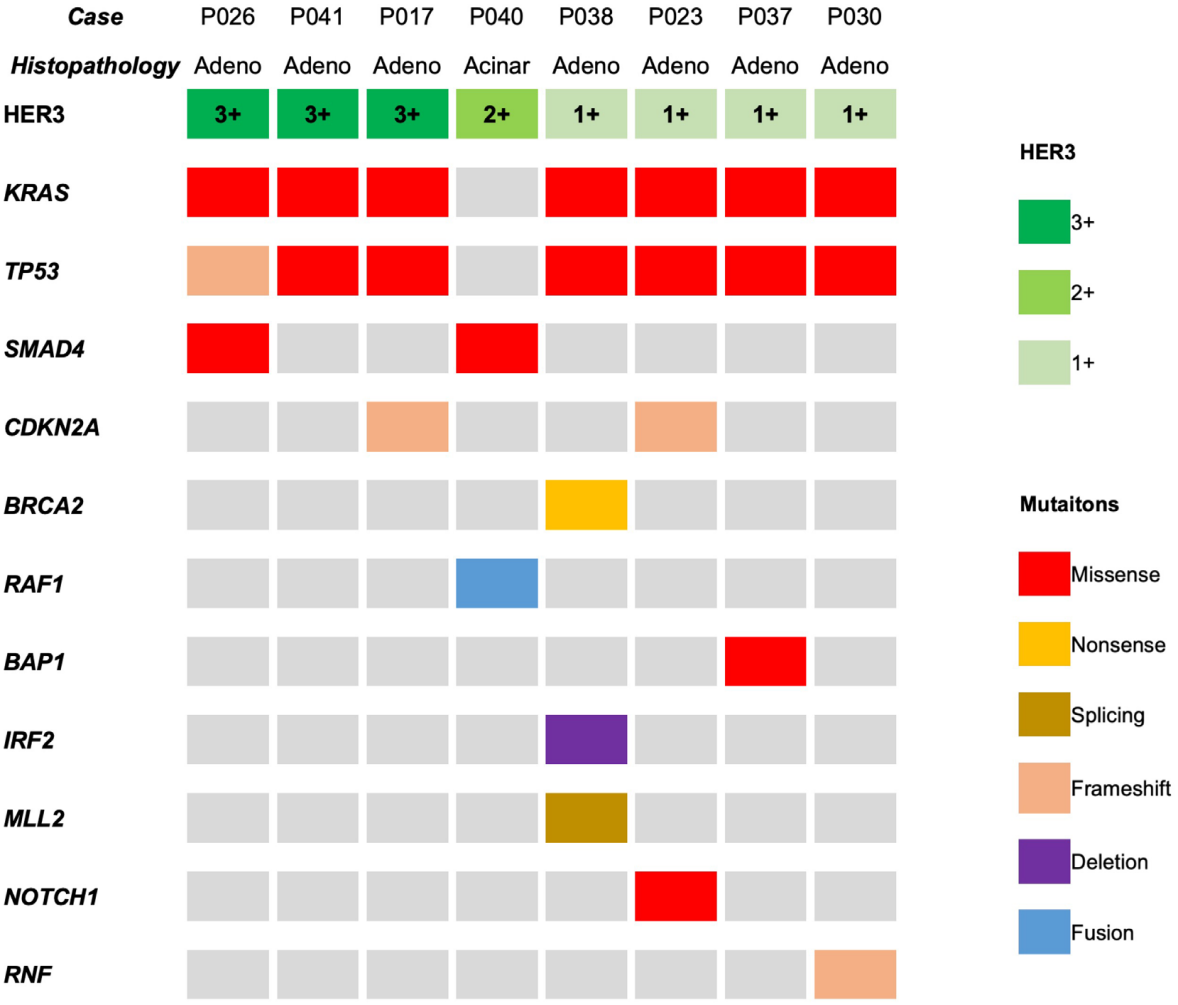
Summary of the genomic alterations and HER3 expression in eight cases. The figure includes only mutations evaluated as pathogenic or likely pathogenic. acinar, acinar cell carcinoma; adeno, adenocarcinoma.

Using the results of the CGP test in eight cases, we examined the association between HER3 expression by IHC and genomic alterations (Figure 4). HER3 expression was observed in all eight cases, with an IHC score of 3+ in three cases, 2+ in one case, and 1+ in four cases. A tissue‐based CGP test was performed in seven patients, five of whom underwent testing with FoundationOne CDx and two with the OncoGuide NCC Oncopanel System, which were performed on the same samples tested for HER3 expression. The remaining patient was tested using the liquid‐based FoundationOne Liquid CDx, which was performed at a different timepoint from tissue collection. No cases of HER3 amplification or mutation were identified. No amplifications or mutations were observed in HER family members EGFR and HER2. Seven of the eight cases were adenocarcinomas, and both *KRAS* and *TP53* mutations were observed in these seven cases. The remaining case was an acinar cell carcinoma that showed a *RAF1* fusion, no *KRAS* or *TP53* mutations, and an IHC score of 2+. All patients had a low tumor mutation burden and showed a microsatellite‐stable status.

## 4 Discussion

In the present study, we report HER3 expression after chemotherapy in pancreatic cancer. Only a few studies have investigated HER3 overexpression in pancreatic cancer [29, 30, 31, 32]. In these previous reports, IHC scores ≥ 1+ for HER3 expression were found in 62% to 94% of cases; specifically, IHC scores of 2+ were found in 17%–31% of cases, and IHC scores of 3+ were found in 9%–24% of cases. When IHC scores of 3+ and 2+ were defined as HER3 overexpression [30, 31], the incidence of HER3 overexpression in the previous studies ranged from 26% to 41%. While these previous studies have addressed HER3 expression at initial diagnosis, to the best of our knowledge, this is the first study that investigates HER3 expression after chemotherapy in pancreatic cancers. In our study, the rates of IHC scores of 2+ and 3+ were 34% and 29%, respectively, and that of HER3 (2+/3+) was 63% in post‐chemotherapy tissue samples. As the antibodies and protocols used for IHC analysis differ among studies, comparisons of HER3 expression rates between studies need to be carefully considered.

Additionally, to the best of our knowledge, this is the first study to compare the changes in HER3 expression before and after chemotherapy in pancreatic cancer. Only one case showed no change in HER3 expression, whereas the remaining four cases showed a change, including one case with a significant change in HER3 expression from an IHC score of 3+ to 0. Although HER3 is not a known resistance mechanism to EGFR kinase inhibitors, EGFR inhibition leads to increased HER3 membrane expression [21]. HER3 expression is associated with resistance to targeted therapies, including HER2 [9, 14, 15], ALK [16], and BRAF [17] inhibitors; hormonal therapies, including tamoxifen [18] and fulvestrant [19]; and chemotherapeutic agents, including paclitaxel [20]. In our study, we assumed that chemotherapy treatment regimens and treatment efficacy may be associated with changes in HER3 expression; however, no consistent relationship was observed in the pre‐ and post‐treatment comparisons. In contrast, all patients who underwent GnP treatment showed an HER3 expression IHC score of 2+/3+. Rabia et al. demonstrated that resistance to gemcitabine is mainly associated with HER2 and HER3 overexpression in preclinical models of pancreatic cancer and in some cells with HER ligand expression [33]. In gemcitabine‐resistant patient‐derived xenograft models of pancreatic cancer, acquired gemcitabine resistance was efficiently overcome by a pan‐HER antibody mixture, which was a cocktail of anti‐EGFR, anti‐HER2, and anti‐HER3 antibodies. Camblin et al. suggested that standard‐of‐care chemotherapy regimens, such as GnP, increase the expression and activation of insulin‐like growth factor receptor 1 and HER3 in pancreatic cancer cells, rendering the cells tolerant to cytotoxic therapies [34]. Although our study is limited because of the small number of cases evaluated, there might be an association between GnP treatment and HER3 expression in our study as well. HER3 expression may change before and after chemotherapy. Although only 19% of specimens were obtained from biopsies in our study, heterogeneity within the tumor may also affect the assessment of HER3 expression, particularly when biopsy specimens are used. A Detailed investigation of whether chemotherapy induces HER3 expression is needed in the future. In clinical practice, the collection of pre‐ and post‐chemotherapy samples may be limited; therefore, preclinical models such as patient‐derived xenograft models may be useful to confirm changes in HER3 expression pre‐ and post‐chemotherapy.

Prognostic significance of HER3 overexpression in pancreatic cancer remains controversial. On the basis of their analysis of 126 resected pancreatic cancer specimens, Hirakawa et al. reported that the median survival time of patients showing HER3 overexpression was 37.2 months, while that of patients with HER3‐negative samples was 58.6 months (*p* = 0.008), and they concluded that HER3 was an independent predictor of poor prognosis based on multivariate survival analysis [31]. In addition, several studies have reported that HER3 overexpression is associated with an advanced tumor stage and shorter postoperative survival in resected pancreatic cancer [30, 32, 35]. However, Kawesha et al. reported that HER3 overexpression was found in 57% (89/157) of resected pancreatic cancers and that HER3 overexpression in cancer cells showed no relationship with patient survival [36]. Using univariate and multivariate analyses, Velde et al. also found no significant association between HER3 overexpression and survival in patients with resectable pancreatic cancer [29]. Our study showed no significant difference in survival between the HER3 (2+/3+) and HER3 (0/1+) groups. These results should be interpreted with caution because they may show some bias related to the patient background characteristics (such as the proportion of patients with resectable and metastatic tumors) in the HER3 (2+/3+) and (0/1+) groups. Although no significant difference was observed in OS between the HER3 (2+/3+) and HER3 (0/1+) patients in the resectable, locally advanced, or metastatic cohorts, the number of cases was very small (Figure S2). Thus, further studies on HER3 expression and its prognosis are needed in a larger number of cases to reach a definite conclusion.

Few reports have evaluated the association between HER3 expression and genomic alterations; therefore, an additional analysis was performed in this study in order to find if HER3 expression is associated with any gene alterations, especially with HER3 amplification, despite the small number of cases with available genomic data. From our analysis, *HER3* amplification was not detected by next‐generation sequencing in any case showing HER3 expression on IHC. *HER3* amplification is a very rare gene alteration found in 0.2% of pancreatic cancers, according to the Center for Cancer Genomics and Advanced Therapeutics (C‐CAT) database, which consists of data from cancer gene panel tests covered by health insurance in Japan [37]. Furthermore, regardless of HER3 expression, a high proportion of *KRAS* and *TP53* mutations were observed, which are commonly observed in typical pancreatic adenocarcinomas. Additionally, similar results were obtained from the profiling of mutations in *SMAD4* and *CDKN2A*, which are frequently observed in patients with regular pancreatic adenocarcinoma. No distinctive genetic mutations related to HER3 expression were found in the current study. Thus, the presence of distinctive genetic mutations associated with HER3 expression remains a subject of future investigation.

Owing to the widespread expression of membrane HER3 in most patients with pancreatic cancer, HER3 is an appealing molecular target for therapeutic interventions. Patritumab deruxtecan (HER3‐DXd; U3‐1402) is an HER3‐directed antibody–drug conjugate composed of patritumab, a cleavable tetrapeptide‐based linker, and a topoisomerase I inhibitor payload (MAAA‐1181a, an exatecan derivative) [38]. In a phase I study of EGFR inhibitor‐resistant, *EGFR*‐mutated NSCLC, patritumab deruxtecan showed a response rate of 39.2% [39]. In another phase II study of *EGFR*‐mutated NSCLC previously treated with EGFR TKI therapy, patritumab deruxtecan showed an objective response rate of 29.8% [40]. Both studies showed no clear association with clinical response to patritumab deruxtecan and HER3 expression. Notably, 3 out of 5 patients with NSCLC harboring *KRAS* or *NRAS* driver mutations responded to patritumab deruxtecan in another trial, despite the small number of cases evaluated [41]. Patritumab deruxtecan also demonstrated preclinical efficacy in colorectal cancer xenografts, showing a dependence on HER3 expression rather than on KRAS mutations [42]. Therapeutic development of compounds targeting HER3, including patritumab deruxtecan, is expected for pancreatic cancer, which is often characterized by *KRAS* mutations.

This study has some limitations. First, the number of cases was limited; therefore caution is needed for the interpretability of the survival analysis. Second, this study was limited to cases with post‐chemotherapy specimens, including resectable cases and those that underwent conversion surgery or had distant metastases. Additionally, when biopsy specimens are used to evaluate HER3 expression, the results of small biopsy specimens may not reflect the overall tumor status in cases with heterogeneous HER3 expression. Multisite biopsies to improve sample availability are needed to resolve these limitations caused by small sample sizes. Finally, we evaluated HER3 expression on the basis of the evaluation of HER2 in gastric cancer, which is a relatively widely used method but is not clearly defined as a criterion for evaluating HER3 expression. Criteria for evaluating HER3 expression are expected to be established in the future.

In conclusion, our study showed a high prevalence of HER3 expression in pancreatic cancer after chemotherapy treatment. The HER3 expression status of pancreatic cancer is of great interest as a therapeutic target, and further research with a larger sample size is warranted in the future.

## Author Contributions

**Tomoyuki Satake:** writing – original draft (equal). **Chigusa Morizane:** writing – original draft (equal). **Mao Okada:** writing – original draft (equal). **Mariko Nishioka:** writing – original draft (equal). **Nobuyoshi Hiraoka:** writing – original draft (equal). **Satoshi Nara:** writing – original draft (equal). **Tomoya Kakegawa:** writing – original draft (equal). **Maki Kobayashi:** writing – original draft (equal). **Kumiko Koyama:** writing – original draft (equal). **Minoru Esaki:** writing – original draft (equal). **Takuji Okusaka:** writing – original draft (equal).

## Ethics Statement

This study was approved by the Institutional Review Board of the National Cancer Center, Tokyo, Japan (Approval Number: 2018–149).

## Consent

Written informed consent was obtained from all participants.

## Conflicts of Interest

Chigusa Morizane has received research funding from Eisai, Yakult Honsha, ONO Pharmaceutical, Taiho Pharmaceutical, J‐Pharma, AstraZeneca, Merck Biopharma, Daiichi Sankyo, Boehringer Ingelheim, Daiichi Sankyo RD Novare, Labcorp, Hitachi, and MSD K.K.; has consulted for Yakult Honsha, MSD K.K., SERVIER, Boehringer Ingelheim, Taiho, Novartis, AstraZeneca, MSD, and Merck Biopharma; and received speaker honoraria from Novartis, Yakult Honsha, SERVIER, Taiho Pharmaceutical, Eisai, MSD K.K., AstraZeneca, TORAY, Guardant, and Myriad Genetics. Takuji Okusaka has received research funding from Chugai Pharmaceutical, Eisai, Novartis Pharma, Bristol‐Myers Squibb Company, AstraZeneca, MSD, Chiome Bioscience, Syneos, Incyte jp, and SYSMEX; has consulted for AstraZeneca, Eisai, Ono Pharmaceutica, FUJIFILM, Toyama Chemical, Daiichi Sankyo, Dainippon Sumitomo Pharma, Nihon Servier, Chugai Pharmaceutical, and Novartis Pharma; received speaker honoraria from AstraZeneca, Syneos, Eisai, Ono Pharmaceutical, Johnson & Johnson, Taiho Pharmaceutical, Chugai Pharmaceutical, Nihon Servier, Novartis Pharma, Myriad Genetics, and Yakult Honsha, and Daiichi Sankyo; and is an editorial board member of Cancer Science. Kumiko Koyama, Tomoya Kakegawa, and Maki Kobayashi are employees of Daiichi Sankyo Co. Ltd. The other authors declare no conflicts of interest.

## Supporting information


**Figure S1.** Patients flow diagram. Overall survival according to HER3 expression and disease stage. (A) Overall survival for resectable stage. (B) Overall survival for locally advanced stage. (C) Overall survival for metastatic stage.

**Table S1.** Clinical and therapeutic details and the outcome of patients included in the study.

Table S2.

## Data Availability

The data that support the findings of this study are available from the corresponding author upon reasonable request.
