# Supplementary figures and images for: Prevalence of HER3 Expression in Pancreatic Cancer Patients Treated With Systemic Chemotherapy
Source: Cancer Med. 2024 Dec 9;13(23):e70474. doi: 10.1002/cam4.70474 (PMC11626478; doi:10.1002/cam4.70474)

Supplementary Figure S1


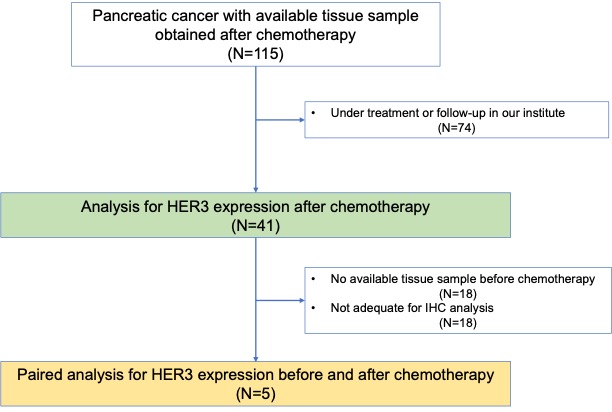


Supplementary Figure S2

(A)


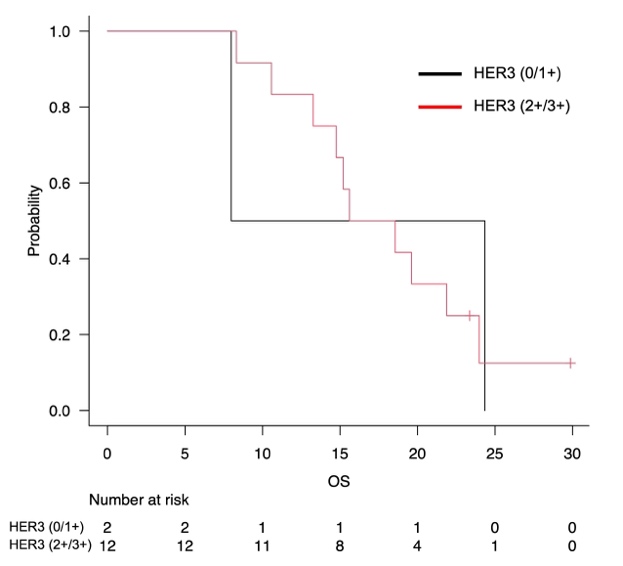


(B)


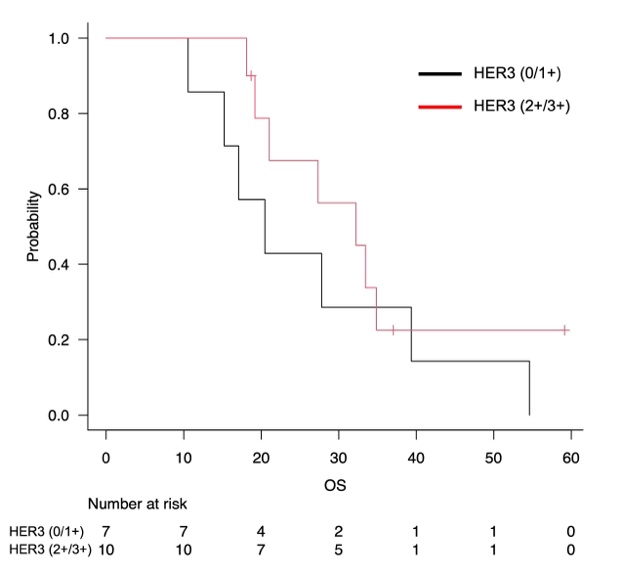


(C)


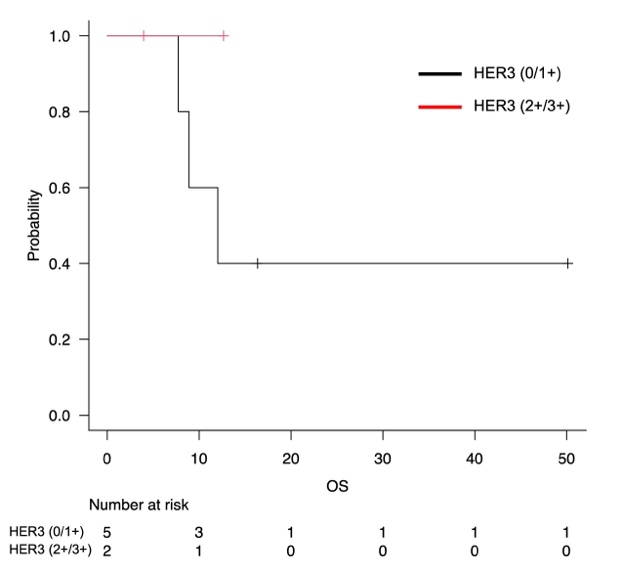

Supplement: Supplementary file 1 — Figure S1. Patients flow diagram. Overall survival according to HER3 expression and disease stage. (A) Overall survival for resectable stage. (B) Overall survival for locally advanced stage. (C) Overall survival for metastatic stage. [file CAM4-13-e70474-s002.docx]
